# Kimura Disease: An Unusual Presentation of Parotid Mass in a Sickle Cell Disease Patient

**DOI:** 10.7759/cureus.24787

**Published:** 2022-05-06

**Authors:** Farhan M Alanazi, Abdulaziz S Alobaid, Rawan M Alahmadi, Fareed AlGhamdi

**Affiliations:** 1 Otolaryngology - Head and Neck Surgery, Prince Mohammed Medical City, Aljouf, SAU; 2 Otolaryngology - Head and Neck Surgery, Prince Sultan Military Medical City, Riyadh, SAU; 3 Otolaryngology - Head and Neck Surgery, Buraydah Central Hospital, Buraydah, SAU

**Keywords:** kd, kimura disease, lymphadenopathy, neck swelling, surgical excision, eosinophilia, partoidectomy, sickle cell, kimura, parotid mass

## Abstract

Kimura disease (KD) is a rare benign chronic inflammatory condition of unknown cause, usually affecting young men of the Asian race. It is frequently associated with nephrotic syndrome. In this report, we present an uncommon case of KD in a 40-year-old Saudi man with sickle cell disease who presented with swelling on the right side of his face. CT scan of the head and neck showed the asymmetrical appearance of both parotid glands: the right side appeared heterogeneously enlarged, with adjacent moderate-to-significant fat stranding. Histologically, hyperplastic changes in lymphoid tissue were observed. The patient underwent superficial parotidectomy and was then followed up till the healing of the surgical site with no complications.

## Introduction

Kimura disease (KD) is a rare benign chronic inflammatory disease of unknown cause, usually involving lymph nodes and the head and neck's deep subcutaneous tissue. It is primarily seen in Asian men, but some non-Asian cases have been reported [1,2]. The common symptoms in patients with KD include a solitary swollen painless lymph node or generalized lymphadenopathy (67-100%), eosinophilia, and increased serum immunoglobulin E (IgE) levels [3]. Histological findings include lymphoid hyperplasia with well-developed lymphoid follicles, eosinophil infiltration, and varying degrees of fibrosis [4]. Worldwide, since the advent of histological diagnosis, slightly more than 200 cases have been documented [5]. Men are more typically affected than women, with a male-to-female ratio of 3:1 [6]. Multiple treatment modalities have been described to manage KD: oral corticosteroids, leflunomide, cyclosporine, intravenous immunoglobulin (IVIG), pentoxifylline, imatinib, radiotherapy, and surgical resection, which is the most commonly used method [1,4,6-12]. In this report, we present an atypical case of KD in a 40-year-old Saudi male who was known to have sickle cell disease.

## Case presentation

**Figure 1:**
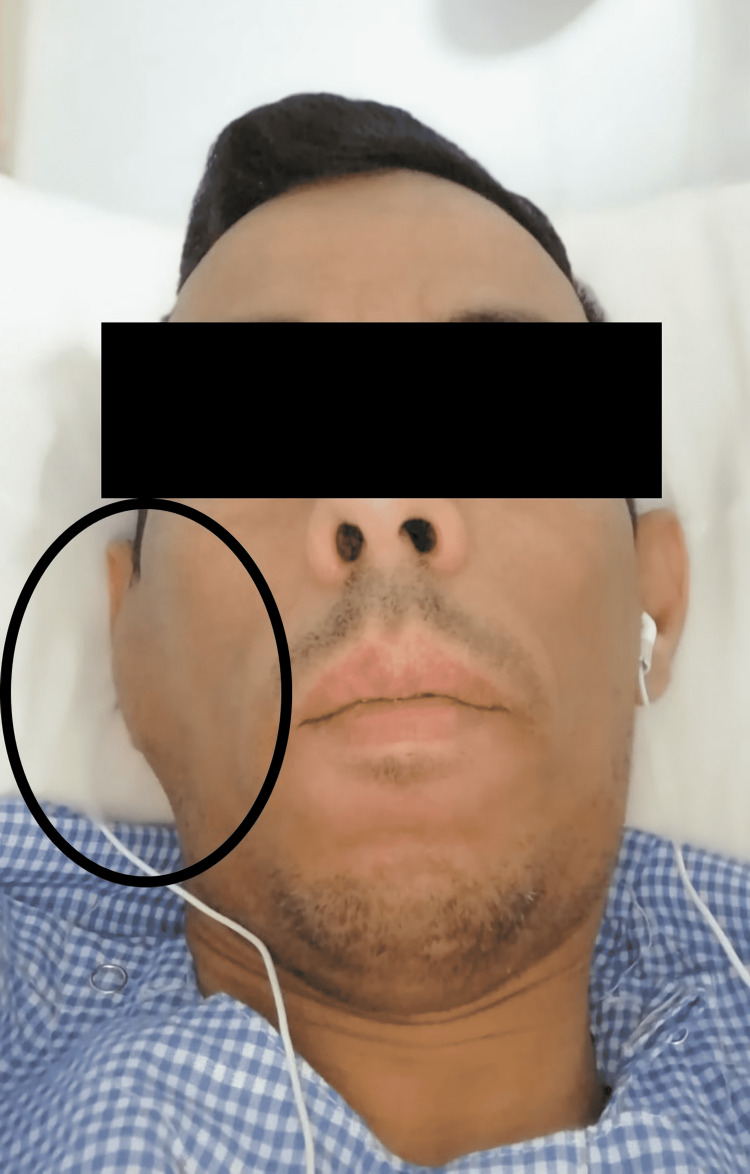
Clinical image showing enlarged right parotid gland

A 40-year-old male was referred to evaluate a painless, firm mass in the right preauricular area that had been slowly growing over the past two years. He denied any history of pain, numbness, or facial weakness. Examination revealed a right pre-auricular nonfixed mass sized 2 x 3 cm (Figure 1). The rest of the assessment of the head and neck was unremarkable. Investigations showed eosinophilia and a high IgE serum level.

**Figure 2:**
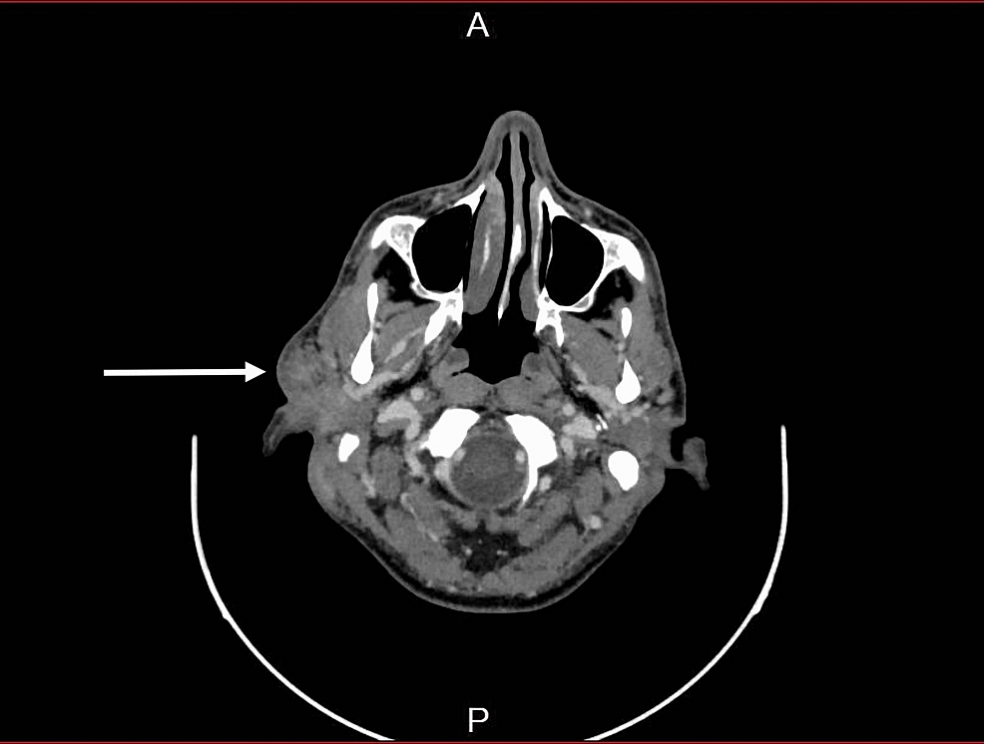
CT showing enlarged heterogeneous right parotid gland CT: computed tomography

**Figure 3:**
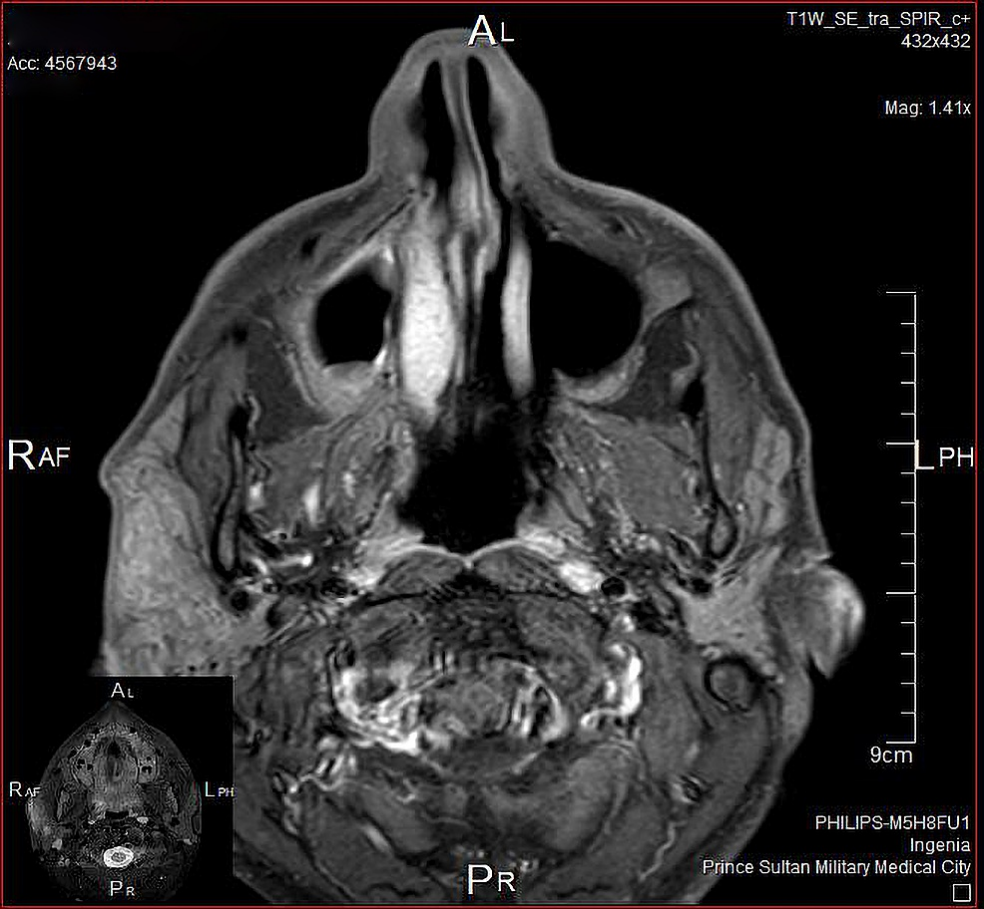
MRI showing infiltration of the overlying subcutaneous tissue - image 1 MRI: magnetic resonance imaging

**Figure 4:**
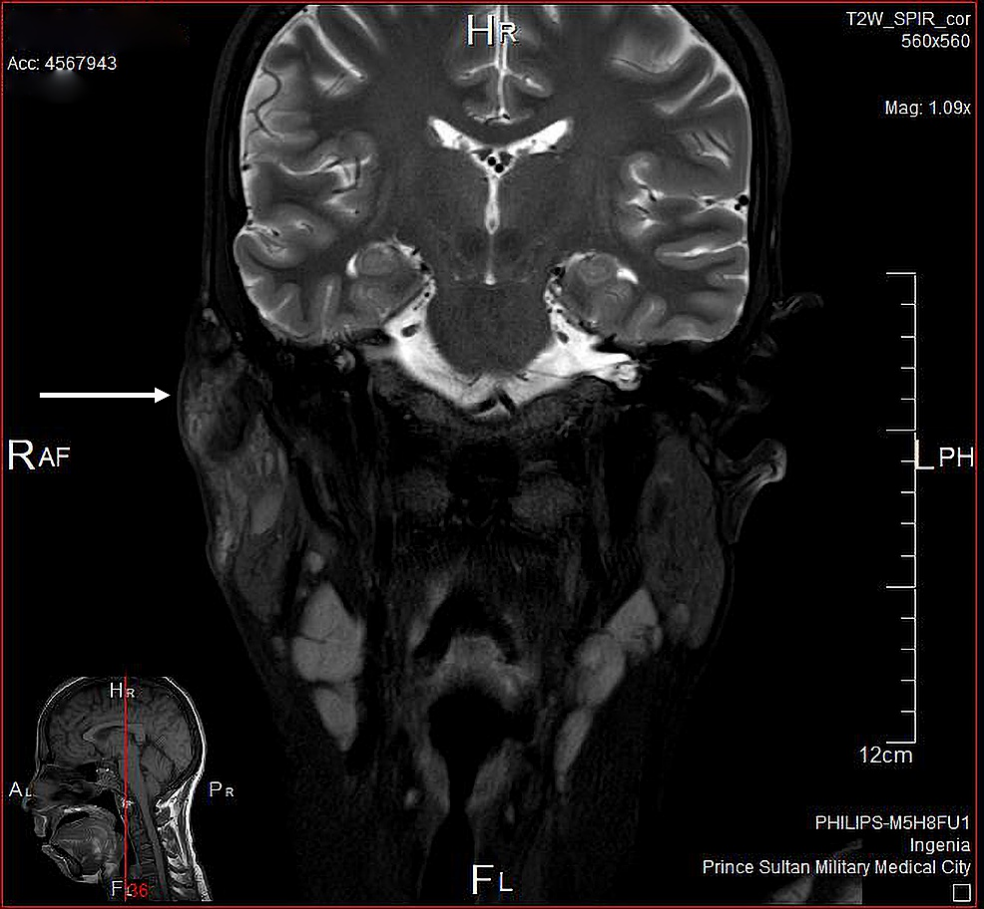
MRI showing infiltration of the overlying subcutaneous tissue - image 2 MRI: magnetic resonance imaging

The patient underwent fine-needle aspiration cytology, which showed lymphoid tissue. CT scan of the head and neck showed the asymmetrical appearance of both parotid glands, as the right side appeared heterogeneous, enlarged with adjacent moderate-to-significant fat stranding (Figure 2). MRI scan of the head and neck showed moderate swelling of the superficial part of the right parotid gland associated with significantly high T2, low T1 signal intensity, and intense enhancement on the post-contrast images. Infiltration of the overlying subcutaneous tissue was noted, yet the cutaneous/skin condition appeared normal (Figures 3, 4).

**Figure 5:**
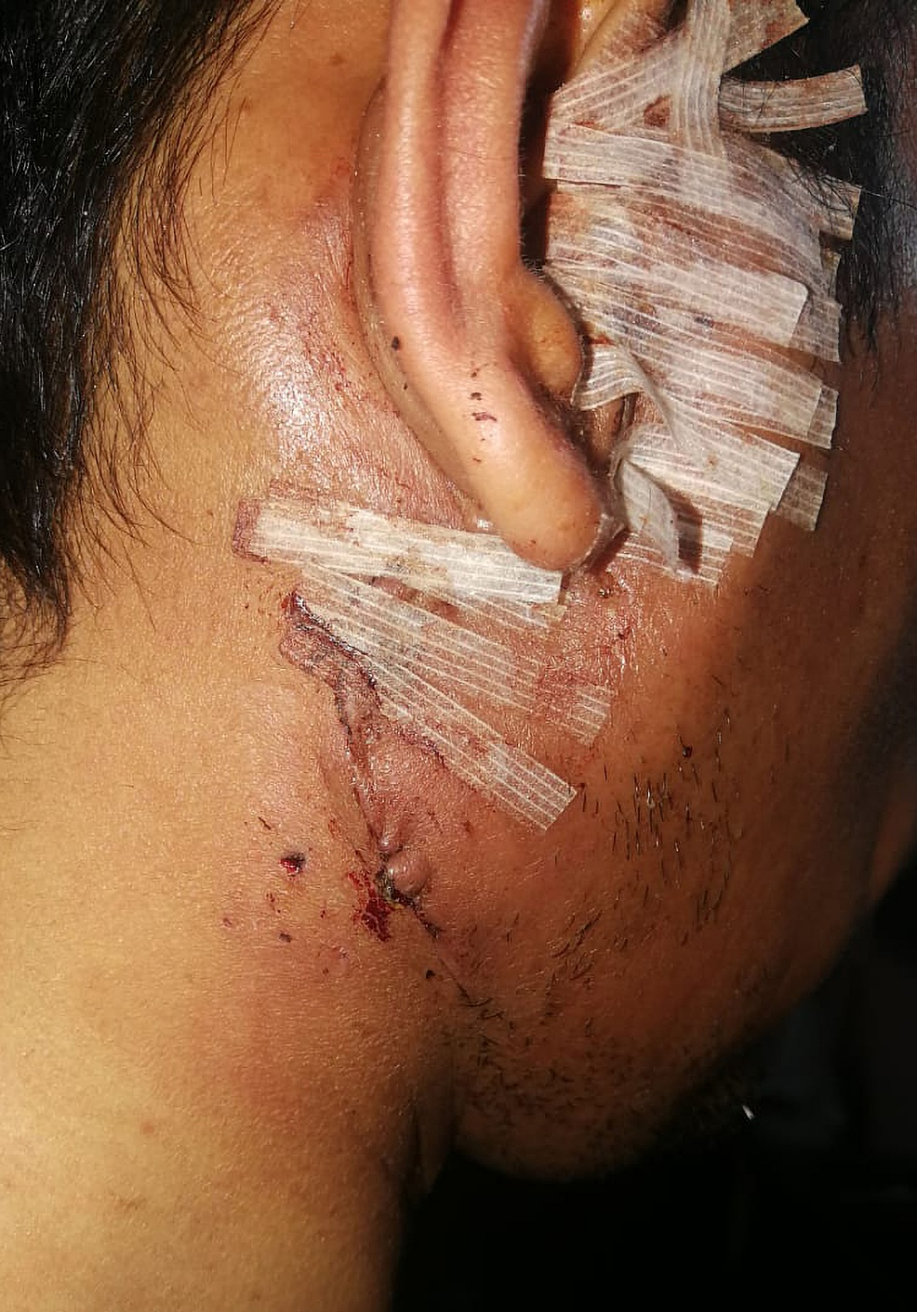
Image on the first postoperative day showing clean wound and intact facial nerve

**Figure 6:**
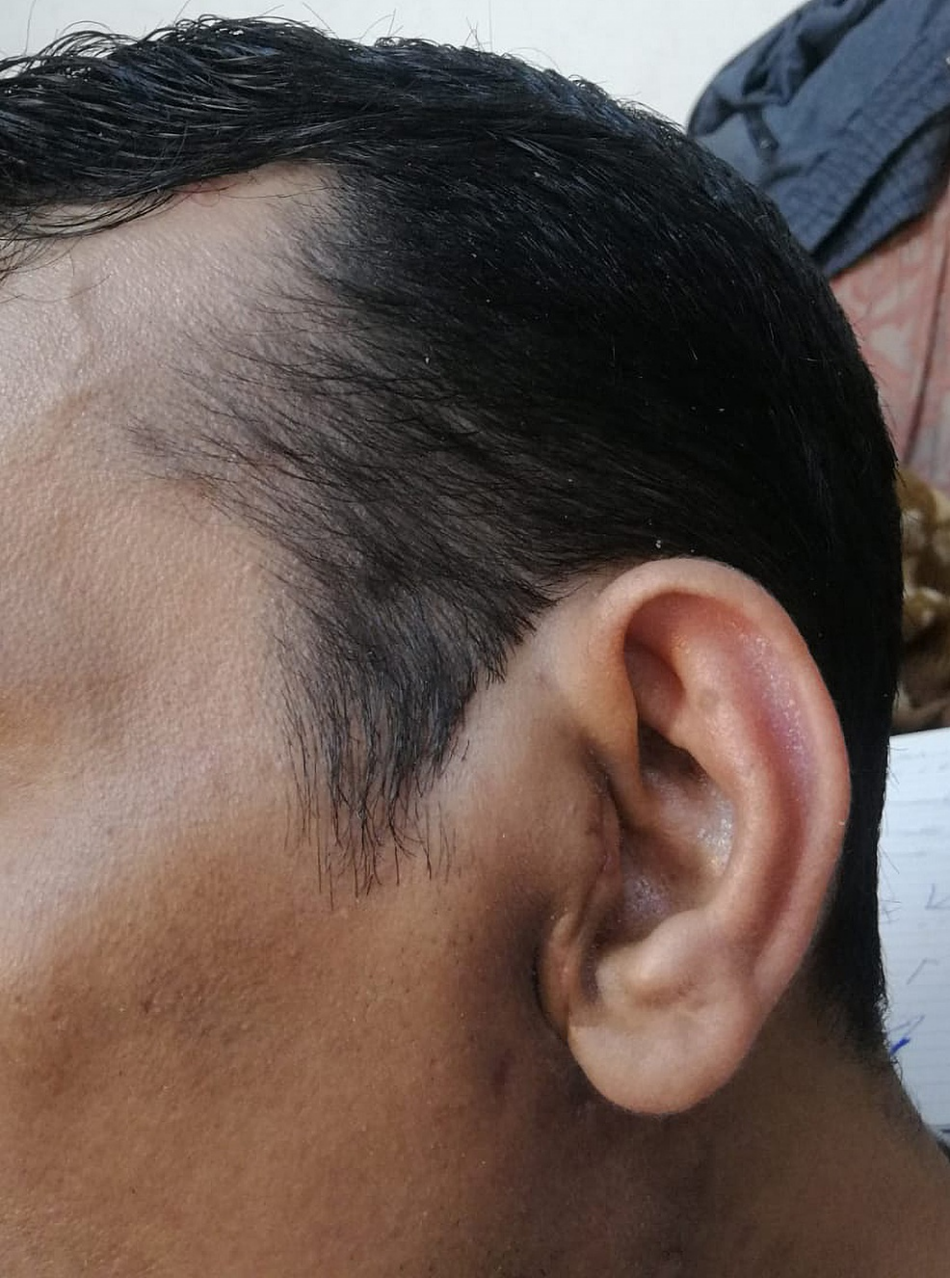
Image at the six-week follow-up showing the healed surgical site

After discussing the modalities for treating such masses and the benefits of the surgery, the patient agreed to undergo a right superficial parotidectomy. On the first postoperative day, he was found to be doing well with a clean wound and intact facial nerve (Figure 5). Histopathology of the mass showed features of Kimura. The patient was kept on regular follow-up in the clinic, and the surgical site healed well in six weeks, as shown in Figure 6.

## Discussion

KD is a benign condition that affects the lymph nodes, subcutaneous tissue, and parotid gland. It was first described in 1948 by Kimura in the literature in Japanese after the first report by Kim and Szeto in the literature in Chinese in 1937 [13]. KD is predominantly seen in middle-aged Asian men. Patients commonly present with a painless firm mass in the head or neck area, with regional lymphadenopathy varying in size [14]. KD has no definitive etiology or pathophysiology; however, it could be a self-limiting allergy or autoimmune reaction triggered by a chronic antigenic stimulus. Also, the disease does not have a specific radiological or cytological feature, but it is characterized by high levels of IgE, eosinophilia, and enlarged painless lymph nodes with the histological findings of preserved lymph node architecture and hyperplastic germinal centers [15]. In more than half of the cases, the disease is accompanied by renal affection, such as nephrotic syndrome and extra membranous glomerulonephritis [13].

The treatment modalities of KD mainly aim to prevent its recurrence, preserve cosmesis, and avoid other systems being affected, such as the renal system. Surgical management is the most popular treatment option, especially in localized cases, and the lowest recurrence rates of KD have been achieved with surgical resection and low-dose postoperative radiation [11,16]. KD has an overall good prognosis with no cases of malignant transformation reported so far; however, renal involvement renders the prognosis unpredictable, depending on the degree of nephropathy. Hence, patients with KD should be monitored for disease recurrence and the onset of renal disease.

## Conclusions

KD is a benign disease that mainly affects Asian men in their forties. It usually involves the head and neck region. The initial presentation may be mistaken for cancer. Enlarged painless lymph nodes, high IgE levels, and eosinophilia are the main characteristics of the disease, and hence when patients present with a head and neck mass and lymphadenopathy, KD should be included in the differential diagnosis and further explored. Histopathology is usually needed for a definitive diagnosis. Surgical treatment may be employed to maintain the cosmetic appearance and avoid recurrence, with medical treatment as another option. KD has an overall good prognosis after conservative management.
